# Polyphenols and Bone Health: A Comprehensive Review of Their Role in Osteoporosis Prevention and Treatment

**DOI:** 10.3390/molecules30214154

**Published:** 2025-10-22

**Authors:** Pasquale Perrone, Chiara De Rosa, Stefania D’Angelo

**Affiliations:** 1 Department of Medical, Human Movement, and Well-Being Sciences, Parthenope University of Naples, 80133 Naples, Italy; 2 Department of Clinical Medicine, School of Medicine and Surgery, University of Naples Federico II, 80131 Naples, Italy; chiara.derosa8@studenti.unina.it

**Keywords:** antioxidants, bone metabolism, osteoblasts, osteoclasts, osteoporosis, polyphenols

## Abstract

Osteoporosis is a progressive bone disorder characterized by decreased bone mineral density and structural deterioration, leading to increased fracture risk. Conventional treatments, although effective, are limited by adverse effects and low long-term adherence. In recent years, polyphenols, plant-derived bioactive compounds, have emerged as promising candidates for bone health promotion due to their antioxidant, anti-inflammatory, and osteo-regulatory properties. This review synthesizes the current preclinical and clinical evidence on the potential of polyphenols, including quercetin, resveratrol, curcumin, isoflavones, and epigallocatechin gallate, to modulate bone metabolism and prevent or mitigate osteoporosis. Mechanistically, polyphenols enhance osteoblastogenesis, inhibit osteoclast differentiation, regulate the RANKL/OPG axis, and activate key osteogenic pathways such as Wnt/β-catenin and MAPKs. Additionally, their estrogen-like activity and ability to modulate gut microbiota offer further therapeutic potential. Preclinical models consistently demonstrate improvements in bone mass, architecture, and turnover markers, while clinical trials, although limited, support their role in preserving bone density, particularly in postmenopausal women. Despite promising outcomes, variability in bioavailability, dosage, and study design limits current translational application. Further large-scale clinical studies and standardized formulations are needed. Polyphenols represent a compelling adjunct or alternative approach in the integrated management of osteoporosis.

## 1. Introduction

Osteoporosis is a systemic skeletal disorder of global clinical relevance, characterized by a progressive reduction in bone mineral density (BMD) and significant alterations in bone tissue microarchitecture. This condition leads to a substantial increase in skeletal fragility and, consequently, a heightened risk of fractures, which frequently occur even after minimal trauma, such as a simple fall, spinal flexion, and lifting a modest weight [1].

The World Health Organization has defined osteoporosis as a true “social disease”, acknowledging the considerable impact this condition exerts on contemporary society. This classification reflects not only its widespread prevalence but also the profound socioeconomic and healthcare-related consequences it imposes on patients, families, and national health systems [2].

In recent decades, several pharmacological strategies have been developed for osteoporosis management. These include antiresorptive agents, such as bisphosphonates and denosumab, which reduce bone turnover and bone resorption, as well as anabolic therapies like teriparatide and abaloparatide, which stimulate new bone formation and improve bone quality [3]. More recently, romosozumab, a monoclonal antibody targeting sclerostin, has been introduced as a dual-acting drug with both anabolic and antiresorptive properties [4]. Hormone replacement therapy (HRT) and selective estrogen receptor modulators (SERMs) also represent therapeutic options, although their clinical use is often restricted due to concerns about long-term safety, including cardiovascular events and cancer risk [5]. These therapies have demonstrated substantial efficacy in reducing vertebral, non-vertebral, and hip fractures, and remain the cornerstone of clinical management for patients at high fracture risk.

While conventional pharmacological therapies are effective, they are often associated with significant limitations, including long-term side effects, poor patient compliance, and economic burdens for healthcare systems [6]. Bisphosphonates, for example, are associated with gastrointestinal intolerance, rare but serious complications such as atypical femoral fractures and osteonecrosis of the jaw, and concerns about long-term skeletal safety. Denosumab discontinuation may lead to a rebound increase in bone turnover and elevated fracture risk, requiring careful management [7,8]. Anabolic therapies like teriparatide and abaloparatide are effective but limited by high costs and restrictions on treatment duration. Even novel therapies such as romosozumab, while promising, raise safety concerns related to potential cardiovascular adverse events [9,10]. These challenges underline the importance of integrating pharmacological and non-pharmacological strategies to optimize long-term outcomes. The increasing interest in alternative or complementary therapeutic approaches to conventional pharmacological treatments stems from the urgent need to identify safe, effective, and sustainable strategies for the prevention and management of osteoporosis.

Natural medicine and phytopharmacology are emerging as promising fields of research, offering innovative approaches based on plant-derived bioactive compounds. These strategies align with the principles of integrative medicine, which aims to combine the best evidence-based practices from conventional medicine with safe and effective complementary therapies, tailoring treatments to the individual characteristics of each patient [11]. Within this context, particular attention has been directed toward polyphenols, a class of natural compounds with notable bioactive properties.

**Figure 1:**
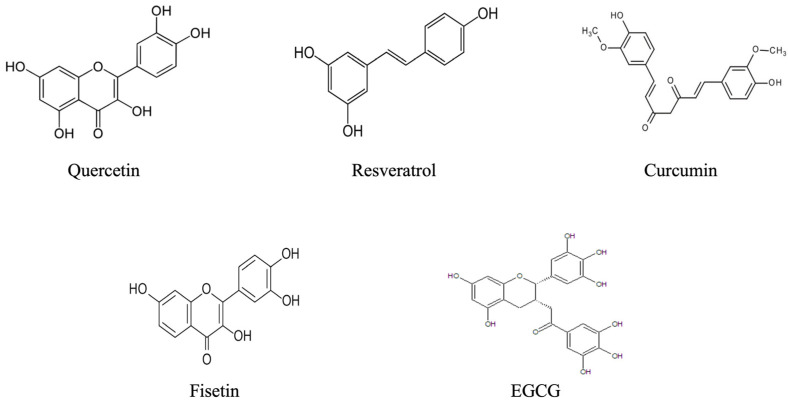
Representation of the main polyphenols discussed in the manuscript. The image highlights the chemical structures and categories of the compounds.

Polyphenols are considered among the most promising classes of natural bioactive compounds for human health. They comprise a vast family of over 8000 naturally occurring organic molecules found in the plant kingdom. Synthesized by plants as specialized secondary metabolites, polyphenols play critical protective roles against external stressors, pathogens, ultraviolet radiation, and environmental challenges [12] (Figure 1).

The Mediterranean diet is recognized as an optimal nutritional model for polyphenol intake, being characterized by a high consumption of fruits, vegetables, whole grains, extra virgin olive oil, and moderate red wine intake during meals [13,14,15]. Epidemiological studies have demonstrated that long-term adherence to polyphenol-rich diets provides protection against the development of various chronic conditions, including cancer, cardiovascular diseases, diabetes, osteoporosis, and neurodegenerative disorders [16,17,18,19].

Unlike previous reviews, which have primarily focused on classical polyphenols and their antioxidant or estrogen-mimicking effects, the present paper provides a comprehensive and updated overview up to 2025, including the most recent preclinical and clinical studies. We emphasized emerging aspects such as the role of the gut–bone axis, microbiota-derived metabolites (e.g., 4-hydroxyphenylacetic acid), and innovative delivery systems such as nanoparticles designed to enhance bioavailability. By adopting a translational perspective, this review integrates molecular mechanisms, in vitro and in vivo data, and clinical findings, with the aim of outlining both the potential and the limitations of polyphenols in osteoporosis management.

The objective of this review is to provide a critical and comprehensive analysis of the current scientific evidence regarding the use of polyphenols in the prevention and treatment of osteoporosis. By synthesizing existing findings, this paper aims to offer a meaningful contribution to the international literature on this emerging topic. This review aligns with the ongoing shift toward personalized and integrative medicine, which increasingly recognizes the role of natural bioactive compounds, such as polyphenols, as key elements in multimodal strategies for managing chronic age-related diseases.

## 2. Pathophysiology of Osteoporosis

Osteoporosis is a complex skeletal disease characterized by a fundamental imbalance in the physiological processes of bone remodeling, resulting in the progressive loss of bone mass and the deterioration of bone tissue microarchitecture. This imbalance reflects a disruption in the dynamic equilibrium between osteoblasts (cells responsible for the synthesis and mineralization of bone matrix) and osteoclasts (cells involved in bone tissue resorption) [20].

Bone tissue undergoes continuous remodeling, a process essential for maintaining structural integrity, repairing microdamage, and adapting to mechanical stress. Bone remodeling consists of two sequential phases: first, bone resorption, mediated by osteoclasts that degrade mineralized bone matrix; followed by bone formation, in which osteoblasts synthesize new organic matrix and promote its mineralization [21]. Under physiological conditions, the activity of these two cell types is tightly regulated, ensuring balanced bone turnover and preservation of bone mass and quality. In osteoporosis, however, osteoclastic activity exceeds osteoblastic activity, leading to net bone loss and increased skeletal fragility [22].

The pathogenesis of osteoporosis is multifactorial, involving a complex network of cellular and molecular signals that regulate the differentiation, activation, and apoptosis of osteoblasts and osteoclasts [23].

Estrogen deficiency is a major pathogenic factor, particularly in postmenopausal women. Estrogens exert a protective effect on bone through multiple mechanisms, notably by suppressing the production of pro-inflammatory cytokines such as tumor necrosis factor-alpha (TNF-α) by T lymphocytes, which would otherwise promote osteoclastogenesis. Estrogens also directly inhibit osteoclast differentiation and activity while supporting osteoblast survival and function. Estrogen deficiency leads to increased production of TNF-α and other osteoclastogenic cytokines, which stimulate Receptor Activator of Nuclear Factor κB Ligand (RANKL)-mediated osteoclast differentiation, thereby enhancing bone resorption [24].

Oxidative stress also plays a critical role in osteoporosis pathogenesis. Elevated levels of intracellular reactive oxygen species (ROS), as observed in ovariectomized animal models and postmenopausal women, directly damage osteoblasts, impairing their proliferative and differentiative capacity. Additionally, ROS enhance osteoclastogenesis by modulating the response of osteoclast precursors to RANKL [25]. A pro-oxidative environment also induces the production of pro-inflammatory cytokines such as interleukin-1 (IL-1), interleukin-6 (IL-6), and interleukin-7 (IL-7), which further promote bone resorption. These combined effects accelerate bone mass loss and compromise skeletal tissue quality [26,27].

Chronic inflammation, characteristic of both aging and estrogen deficiency, significantly contributes to osteoporosis development. Activation of the NLRP3 inflammasome (NOD-like receptor family pyrin domain containing 3) triggers hypersecretion of inflammatory mediators such as IL-1β and IL-18, which inhibit osteoblast differentiation and function, leading to cellular dysfunction and apoptosis [28]. Simultaneously, they enhance osteoclast activity, accelerating bone resorption and fostering a local pro-inflammatory environment that perpetuates bone damage. Chronic activation of this inflammatory system is a key mechanism underlying bone loss associated with aging and menopause [29].

Another pivotal molecular mechanism in osteoporosis is the dysregulation of the RANKL/RANK/OPG system, which is central to osteoclastogenesis regulation. RANKL is a transmembrane protein expressed by osteoblasts and other stromal cells that binds to the RANK receptor on osteoclast precursors, promoting their differentiation into mature, active osteoclasts [30]. Osteoprotegerin (OPG), a soluble decoy receptor produced by osteoblasts, binds RANKL, preventing its interaction with RANK and thereby inhibiting osteoclast formation and activity. In estrogen-deficient states, OPG expression decreases while RANKL expression increases, resulting in an imbalance that favors osteoclast activation and excessive bone resorption [31,32].

It is important to note that additional mechanisms contribute to the development of osteoporosis. Alterations in the immune system, particularly increased production of osteoclastogenic cytokines by T lymphocytes and macrophages, play a key role [33]. Mitochondrial dysfunction and metabolic alterations in bone cells also contribute to pathogenesis [34]. Moreover, epigenetic modifications can affect gene expression in both osteoblasts and osteoclasts. Hormonal factors such as calcium, vitamin D, parathyroid hormone (PTH), and calcitonin actively modulate bone metabolism [35].

**Figure 2:**
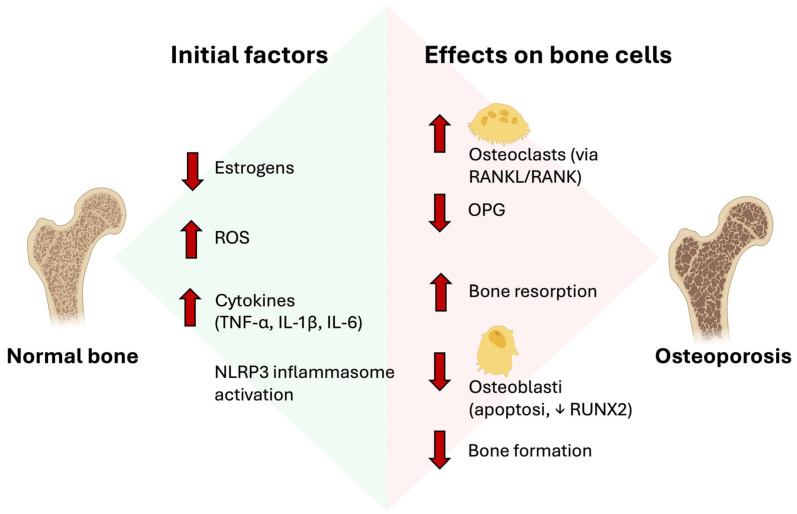
Molecular mechanisms of osteoporosis.

In summary, osteoporosis results from a complex interplay of cellular, molecular, and hormonal factors that disrupt the delicate balance between bone formation and resorption, leading to a predominance of osteoclastic activity and consequent loss of bone mass and quality (Figure 2). Understanding these mechanisms is fundamental for the development of targeted therapeutic strategies, including the use of natural compounds such as polyphenols, which have the potential to modulate the key pathways involved in disease pathophysiology.

Highlighting the clinical and societal relevance of this condition, global epidemiological data reveal the dramatic scale of the osteoporosis burden. According to the most recent estimates, approximately 500 million individuals worldwide (6.4% of men and 21.2% of women aged 50 and above) are affected. Each year, up to 37 million fragility fractures occur in individuals aged 55 and older, equivalent to roughly 70 fractures per minute [36].

Osteoporotic fractures, particularly hip fractures, have devastating consequences for patients. The mortality rate associated with femoral fractures is 15–25% within the first six months, while more than half of patients experience functional disability within the first year post-fracture. Only 30–40% of individuals regain independent performance of daily activities, resulting in a dramatic decline in autonomy and quality of life [37]. For these reasons, current scientific research is increasingly focused on identifying natural compounds capable of preventing bone loss and enhancing the efficacy of existing pharmacological therapies, with the ultimate goal of reducing the incidence and severity of these debilitating events.

## 3. Polyphenols: Classification and Mechanisms of Action

Polyphenols represent a large and diverse class of phytochemical compounds naturally found in a wide variety of plant-based foods, including fruits, vegetables, tea, coffee, red wine, cocoa, and whole grains. Chemically, polyphenols are characterized by the presence of multiple phenolic groups and are traditionally classified into four main categories: flavonoids, phenolic acids, stilbenes, and lignans [38,39].

Flavonoids, the most abundant class, include several subgroups such as flavonols, flavones, flavanones, isoflavones, anthocyanins, and flavanols, each with specific dietary sources and unique biochemical properties [40]. Phenolic acids comprise derivatives of benzoic and cinnamic acids, while stilbenes, including resveratrol, and lignans, found in seeds and cereals, play a significant role in modulating various physiological processes (Table 1) [41].

Beyond their structural diversity, polyphenols are distinguished by their ability to interact with multiple biological pathways, conferring a broad spectrum of biological activities that extend well beyond their well-known antioxidant properties [42]. Numerous epidemiological and experimental studies have demonstrated the beneficial effects of polyphenols on human health, primarily due to their antioxidant, anti-inflammatory, and cell signaling modulatory activities. These compounds can neutralize ROS, protect cellular membranes from oxidative stress, and regulate the expression of genes involved in inflammatory and metabolic pathways [43,44,45].

Their anti-inflammatory effects, in particular, are mediated through the inhibition of key transcription factors such as NF-κB and AP-1, which control the production of pro-inflammatory cytokines [46]. By modulating these pathways, polyphenols contribute to the reduction in chronic inflammation, a central feature of many degenerative diseases. Furthermore, polyphenols interact with the gut microbiota, supporting host homeostasis and positively influencing cardiovascular, neurological, and metabolic health [47,48].

This interaction with the intestinal microbiota is of special interest, as polyphenols can be metabolized by specific bacterial strains into bioactive metabolites, which in turn modulate the composition and functionality of the gut flora. This creates a virtuous cycle that promotes systemic health [49,50]. Clinical studies suggest that a high dietary intake of polyphenols is associated with a reduced incidence of chronic degenerative diseases, including cardiovascular diseases, type 2 diabetes, certain types of cancer, and neurodegenerative disorders [51,52,53,54].

Despite these promising effects, the efficacy of polyphenols in humans is significantly influenced by factors such as their bioavailability, which is often limited due to poor solubility, rapid metabolism, and elimination, as well as individual variability in intestinal absorption and metabolism. Moreover, interactions with other dietary components may modulate both the absorption and biological activity of polyphenols, complicating the assessment of their true therapeutic potential [55,56].

Additional challenges include their generally low permeability across the blood–brain barrier and the risk of toxicity at high concentrations or with prolonged use. Therefore, while polyphenols are considered relatively safe, these pharmacokinetic and safety limitations require further validation through well-designed clinical trials.

These considerations highlight the need for further research aimed at improving the understanding of underlying molecular mechanisms, developing novel formulations to enhance their bioavailability, and designing rigorous clinical protocols to evaluate their efficacy in specific pathological contexts. Only through a multidisciplinary approach will it be possible to fully harness the therapeutic potential of polyphenols and integrate them effectively into prevention and treatment strategies for numerous chronic diseases, including osteoporosis.

### Mechanisms of Action Relevant to Bone Health

Polyphenols exert their beneficial effects on bone tissue through multiple and interconnected molecular mechanisms that act synergistically to promote bone health and regeneration. A central pillar of their activity is their antioxidant effect, which plays a critical role in protecting bone tissue from oxidative stress, a key factor in the pathogenesis of osteoporosis. Polyphenols can donate hydrogen atoms or electrons to neutralize free radicals, thereby preventing the formation of ROS that damage cell membranes, proteins, and DNA in bone cells. By stabilizing cellular membranes, polyphenols specifically protect osteoblasts from oxidative damage that would otherwise impair their function and survival [57,58].

Additionally, reducing ROS levels helps prevent excessive osteoclastogenesis, as free radicals are known to enhance the differentiation and activity of osteoclasts, the bone-resorbing cells. Notably, compounds such as quercetin and resveratrol have been shown to increase the activity of superoxide dismutase (SOD) and catalase, key enzymes in ROS neutralization, thereby reinforcing endogenous antioxidant defenses [59].

Concurrently, polyphenols demonstrate potent anti-inflammatory properties, which are particularly relevant in osteoporosis, a condition characterized by chronic low-grade inflammation. These compounds modulate the expression and secretion of several pro-inflammatory cytokines, including TNF-α, IL-1β, IL-6 and IL-8, which are involved in osteoclast activation and recruitment [60]. Inhibition of these mediators occurs through the modulation of key signaling pathways such as nuclear factor kappa B (NF-κB) and activator protein 1 (AP-1), both of which regulate the gene expression of numerous inflammatory and osteoclastogenic factors. Through this action, polyphenols reduce local inflammation within the bone microenvironment, helping to restore bone turnover in favor of formation over resorption [61].

In vitro studies have also shown that epigallocatechin gallate (EGCG), the primary catechin in green tea, can significantly reduce levels of prostaglandin E2, a pro-inflammatory mediator heavily implicated in bone resorption [62].

Another key mechanism by which polyphenols influence bone metabolism is the modulation of osteogenic signaling pathways, particularly the Wnt/β-catenin pathway, which is essential for the differentiation of mesenchymal stem cells (MSC) into osteoblasts [63,64]. Polyphenols promote the activation of this pathway by enhancing the stability and nuclear translocation of β-catenin, which serves as a transcriptional cofactor for the expression of osteogenic genes. This process stimulates osteoblast proliferation and maturation, supporting the synthesis and mineralization of bone matrix [65].

Additionally, polyphenols help regulate the RANKL/OPG system, a critical axis in osteoclastogenesis. By increasing the expression of OPG, polyphenols reduce the activation of NF-κB signaling in osteoclasts, limiting bone resorption and contributing to the maintenance of bone mass. Emerging evidence also suggests that the effect of polyphenols on the RANKL/OPG system can be enhanced in the presence of vitamin D, pointing to a potential nutritional synergy with therapeutic implications [66].

Polyphenols further modulate the expression of key transcription factors involved in osteogenesis, such as Runt-related transcription factor 2 (RUNX2) and Osterix (OSX), which are considered master regulators of osteoblast differentiation. The upregulation of these factors promotes osteoblast maturation and the production of bone matrix proteins, including type I collagen, osteocalcin, and osteonectin, which are essential for the formation of healthy, functional bone. In experimental models, polyphenol-induced RUNX2 expression has also been associated with increased levels of alkaline phosphatase (ALP), a well-established early marker of osteogenesis [67,68].

A particularly noteworthy aspect is the estrogen-like activity of certain polyphenols, especially isoflavones, which share structural similarity with 17β-estradiol and can bind to estrogen receptors (ERα and ERβ) expressed on osteoblasts. This interaction allows polyphenols to function as phytoestrogens, exerting selective estrogen receptor modulator-like effects that stimulate the differentiation and maturation of bone-forming cells. Activation of estrogen receptors leads to the expression of osteogenic genes such as bone morphogenetic protein 6 (BMP-6), type I collagen, and osteocalcin, thereby enhancing bone formation and counteracting the detrimental effects of estrogen deficiency typical of menopause [69,70].

Moreover, polyphenols regulate several other signaling pathways critical to bone remodeling, including the Mitogen-Activated Protein Kinases (MAPKs), namely ERK1/2, p38, and JNK, which govern the proliferation, differentiation, and apoptosis of both osteoblasts and osteoclasts. Polyphenols modulate the expression and phosphorylation of these kinases, favoring osteoblast activity while inhibiting osteoclast-mediated bone resorption. In parallel, the Insulin-like Growth Factor-1 pathway, when stimulated by polyphenols, promotes osteoblast proliferation and maturation, thereby contributing to the maintenance of bone mass [71].

Finally, polyphenols enhance the expression of BMP, particularly BMP-2, which are essential growth factors for the initiation of osteogenesis. BMP-2 activates intracellular signaling cascades leading to the transcription of osteogenic genes, facilitating the differentiation of MSC into osteoblasts and supporting the formation of bone matrix. The combined action of polyphenols on BMP-2 and Wnt/β-catenin pathways appears to be synergistic, suggesting a network of interactions that amplifies the pro-osteogenic effects of these compounds [72].

In summary, polyphenols act at multiple molecular levels, combining antioxidant and anti-inflammatory activities with the modulation of key pathways involved in bone formation and resorption regulation. This multifactorial and integrated approach supports the use of polyphenols in the protection and enhancement of bone health. Collectively, these findings provide a scientific foundation for the development of nutraceutical and therapeutic strategies based on polyphenols, with potential applications in both the prevention and treatment of osteoporosis and other degenerative bone diseases (Table 2).

## 4. Methodology

**Figure 3:**
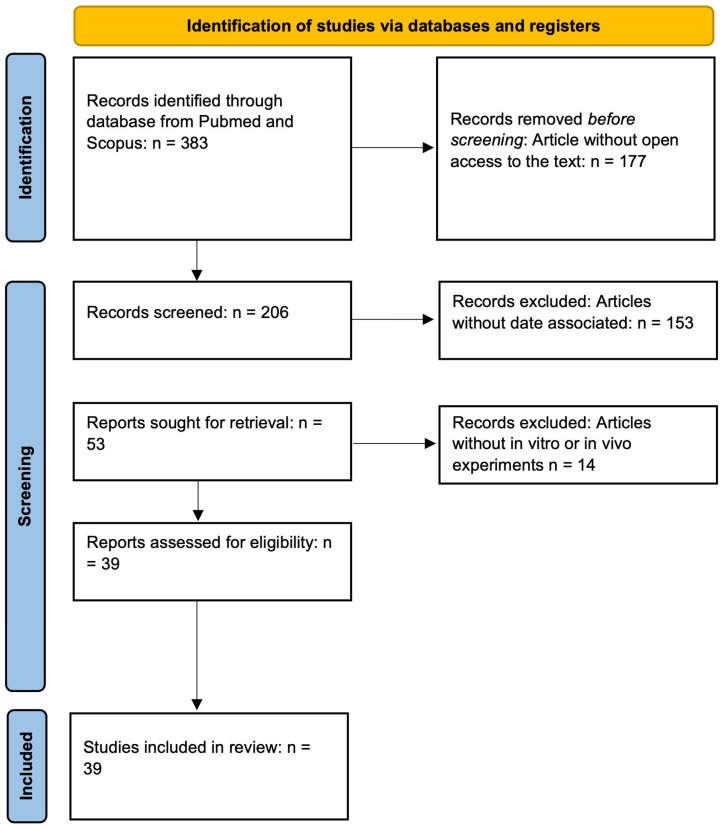
PRISMA flow diagram.

A narrative review of the literature on the effect of polyphenol supplementation on improving bone tissue health was conducted. The databases consulted included SCOPUS, Google Scholar, and PubMed (MEDLINE). Articles were selected based on title, year of publication, review of abstracts, and reading of the full text to assess their relevance. The keywords used in the search included a combination of the terms: “polyphenols,” “osteoporosis,” and “bone tissue.” Articles without access to the full text were not included in the final analysis. Studies were included and selected based on their relevance in associating improved bone health related to the use of polyphenols in cellular, animal, and human models (Figure 3).

## 5. Preclinical Evidence (In Vitro and In Vivo)

### 5.1. In Vitro Studies on Osteoblastic/Osteoclastic Cells

Preclinical evidence regarding the efficacy of polyphenols in modulating bone metabolism is widely supported by numerous in vitro and in vivo studies that have explored the cellular and molecular mechanisms underlying their beneficial effects. These studies have used cellular models of osteoblasts and osteoclasts, as well as animal models of osteoporosis, to evaluate how polyphenols influence bone formation and bone resorption. In the context of cellular investigations, particular attention has been paid to the effect of polyphenols on the differentiation and functionality of osteoblasts, cells fundamental to bone formation (Table 3). Among the most studied polyphenols, quercetin has attracted particular interest for its ability to modulate osteoblast activity. Several studies have investigated its biological properties, outlining a complex and potentially therapeutic profile of action.

#### 5.1.1. Quercetin

Regarding in vitro studies on osteoblastic cells, quercetin emerges as one of the most studied polyphenols. At concentrations of 50 μM, quercetin has been shown to significantly stimulate ALP activity, an early marker of osteoblast differentiation, through activation of the ERK pathway, which is activated downstream of the estrogen receptor. This suggests an estrogen-like mechanism favoring osteoblast maturation [73]. Additionally, at lower concentrations (10 μM), quercetin promotes osteoblast differentiation by stimulating the expression of key factors such as TGF-β1, BMP-2, and RUNX2 through the coordinated activation of MAPKs (ERK1/2, p38, and JNK), essential for regulating cellular proliferation and differentiation [74]. These results highlight the dose-dependent ability of quercetin to favorably modulate osteoblast biology, opening interesting therapeutic perspectives.

Further experimental evidence confirms quercetin’s selectivity towards specific cell types, showing a potentially safe profile with respect to healthy cells. In a study by Lezcano et al., quercetin (1 μM, 48 h) significantly increased the number and viability of healthy human osteoblasts. Moreover, wound healing and cell adhesion assays demonstrated that 1 μM quercetin significantly stimulated both parameters in osteoblasts. Interestingly, at higher concentrations quercetin (20–100 μM) instead reduced the number and viability of osteoblastic tumor cells. Overall, these results indicate that low concentrations of quercetin stimulate osteoblastogenesis but have no effect on tumor osteoblast growth, for which only high concentrations are effective [75]. These observations suggest a selective action of quercetin, capable of stimulating normal cells and inhibiting tumor cells depending on the concentration used.

In addition to direct effects on cell proliferation and differentiation, quercetin also exhibits protective properties against oxidative stress, a known factor that impairs bone homeostasis. Smokers often suffer from compromised fracture healing, often due to poor bone quality and stability. Cigarette smoke damages bone cells and their homeostasis because of increased ROS formation. The aim of the study by Braun et al. was to verify whether quercetin could protect osteoblasts from the toxic effects of smoking. Results showed that quercetin improved their viability by increasing the expression of antioxidant enzymes heme oxygenase and superoxide dismutase [76]. This highlights the role of quercetin as a potential antioxidant protective agent under oxidative stress conditions induced by environmental factors.

Even in the context of bacterial inflammatory diseases, quercetin has demonstrated significant efficacy in preserving osteoblast functionality. In a study by Wang et al., quercetin reversed the inhibition of osteoblast differentiation induced by LPS via MAPK signaling. These results suggest that quercetin could potentially be useful as a therapeutic agent to restore osteoblast function even in bone diseases induced by bacteria. This ability to counteract bacterial inflammatory action strengthens the hypothesis of using quercetin also in osteolytic conditions of infectious origin [77].

An innovative approach to improve the clinical efficacy of quercetin is represented by its integration with biomaterials and scaffolds for bone regeneration. In this regard, in an elegant study by Song et al., the authors designed a silk fibroin/hydroxyapatite scaffold embedded with quercetin at various concentrations to promote osteogenesis, focusing particularly on quercetin’s capacity to enhance bone health. The demand for bone grafts to treat bone defects is steadily increasing worldwide due to the continuous rise in conditions like injuries, trauma, diseases, and infections. Results highlight the efficacy of quercetin for bone regeneration. It is, therefore, hypothesized that such synthesized scaffolds provide a bone-like biomimetic microenvironment, promoting osteoblast differentiation and suggesting a potential high-performance alternative graft for bone regeneration [78]. The combination of advanced biomaterials and bioactive natural molecules represents an innovative strategy for bone regeneration.

In parallel, several studies have explored quercetin’s role in complex pathological contexts such as osteoporosis secondary to mineral metabolism disorders. In 2023, a study reported the mechanism of action of quercetin in treating osteoporosis induced by iron overload. In vitro, quercetin increased ALP activity in MC3T3-E1 cells in an iron overload environment, promoted formation of mineralized bone nodules, and increased expression of Runx2 and Osterix. Moreover, quercetin was able to reduce FAC-induced apoptosis and ROS production by decreasing Caspase3 and Bax expression and increasing Bcl-2 expression. Even under pathological conditions associated with mineral metabolism disorders, quercetin shows osteoprotective effects confirmed at the molecular level [79].

A study by Pang et al. aimed to investigate the overall effect of quercetin on the proliferation of mouse bone marrow MSC and their osteogenic differentiation in vitro. Data showed that quercetin promotes proliferation and osteogenic differentiation of MSC. Quercetin enhances activation of BMP signaling pathways and increases expression of downstream genes such as OSX, RUNX2, and OPN through the endoplasmic reticulum. The concerted activation of osteogenic signaling pathways confirms the versatility of quercetin as a modulator of MSC biology [80]. Quercetin’s ability to modulate MSC further expands its potential in regenerative medicine.

These data clarify that quercetin induces osteogenesis; however, its solubility, stability, and bioavailability limit its therapeutic use. Therefore, in a study by Sharma et al., selenium nanoparticle-based quercetin nanoformulations were synthesized and their osteogenic stimulation and localized bone regeneration capacity were evaluated. Data showed these nanoparticles can restore bone remodeling and, when incorporated into hydrogels, enhance cellular uptake and bioavailability of quercetin in clinical settings, enabling innovative orthopedic and regenerative therapies for bone loss/defects [81]. The use of nanotechnologies proves fundamental to overcoming pharmacokinetic barriers and making polyphenols clinically applicable.

Even the metabolism of quercetin, particularly its plasma metabolites, can influence the antioxidant response of bone cells, a crucial aspect for osteoporosis prevention. Oxidative stress contributes to osteoporosis by suppressing osteoblast differentiation, suggesting antioxidant responses in osteoblasts may be a valid preventive strategy. In this context, plasma metabolites of quercetin have been studied to understand their effects on osteoblast antioxidant responses. Quercetin increases the antioxidant response in many cell types, but further studies are needed to understand the effects of quercetin plasma metabolites on osteoblast antioxidant response. Consequently, a 2015 study aimed to examine antioxidant response genes and proteins in osteoblasts exposed to quercetin plasma metabolites. Osteoblasts isolated from fetal rat calvaria were treated with doses up to 20 μM of three different quercetin metabolites present in blood plasma after consumption of quercetin-rich foods or supplements. Upregulation of HO-1 and GCLC was associated with suppression of phosphorylated ERK1/2 and NFκB, but no changes were observed in protein levels of Nrf2. This study demonstrates that the antioxidant response of osteoblasts is differentially stimulated by quercetin metabolites. No synergistic effects of the metabolites were observed [82].

Despite ample positive evidence, it is important to underline that some studies have reported contrary effects, especially at high doses. A 2016 study analyzed the effect of quercetin on MSC differentiation into osteoblasts. Results showed that quercetin (10 μM) inhibited cell proliferation, ALP activity, and mineralization, reducing the expression of ALPL, type I collagen alpha 1, and osteocalcin-related osteoblastogenesis genes in MSCs undergoing differentiation into osteoblasts. Quercetin did not influence proliferation but increased adipogenesis, mainly at a concentration of 10 μM in MSC induced to differentiate into adipocytes. In conclusion, quercetin supplementation inhibited osteoblast differentiation and promoted adipogenesis at the highest tested concentration. The authors therefore suggest such possible adverse effects of high quercetin concentrations should be considered in nutraceutical or pharmaceutical strategies using this flavonol [83]. These data are consistent with another study showing how quercetin inhibited cell viability in a dose- and time-dependent manner in osteoblastic cells. Additionally, similar cytotoxicity of quercetin was observed in stromal cells derived from adipose tissue. However, in that study quercetin also exerted a protective effect against hydrogen peroxide-induced cell death. It is therefore important to keep in mind that data are still complex to obtain a clear answer. Quercetin can exert both protective and deleterious effects in bone repair [84]. These data suggest that quercetin’s effect is highly dose-dependent, with potential implications for safety in therapeutic use.

#### 5.1.2. Resveratrol

Beyond quercetin, other polyphenols have attracted considerable interest for their osteoprotective properties. Among these, resveratrol, curcumin, isoflavones, and compounds derived from medicinal plants are emerging as promising candidates for the treatment and prevention of osteoporosis.

Resveratrol, a stilbene polyphenol found in grapes and red wine, has demonstrated notable effects on osteoblastic cells. Similar to quercetin, resveratrol acts on molecular pathways involved in osteogenesis, including MDM2/p53 and NF-κB/β-catenin, exerting a protective effect on osteoblastic differentiation. Several studies have explored both resveratrol and its precursors or derivatives, highlighting how these compounds favorably modulate MSC differentiation. In a study by Di Benedetto et al., the authors investigated how resveratrol and polydatin, a natural precursor of resveratrol, influence MSC differentiation and consequently bone formation. The results showed that polydatin enhances the osteogenic differentiation of MSC, sharing similar properties with resveratrol. These findings encourage further investigation into the effects of this molecule on bone health and its associated mechanisms of action, with the hope of future effective use in the prevention and therapy of bone loss [85].

At the molecular level, involvement of the MDM2/p53 pathway has been identified as a central hub in resveratrol’s action on osteogenesis. In a 2020 study, KEGG pathway enrichment analysis of resveratrol target genes identified 33 associated pathways, 12 of which were implicated in osteoporosis. In particular, the MDM2/p53 signaling pathway was identified as a potential key pathway among shared pathways. In vitro experiments showed that MDM2-mediated degradation of p53 induced osteoblast differentiation, and resveratrol could partially reverse p53-dependent inhibition of osteogenic differentiation. These results suggest that resveratrol may alleviate osteoporosis at least in part by modulating the MDM2/p53 signaling pathway [86].

Resveratrol also shows efficacy under pro-inflammatory conditions, where cytokines can impair osteoblastic function. The study by Chen et al. aimed to investigate the protective effect of resveratrol against TNF-α-induced inhibition of osteogenic differentiation, thereby alleviating osteoporosis progression. After osteogenic differentiation in MSCs induced with TNF-α, cell viability and calcification capacity were markedly reduced and upregulated by resveratrol treatment. Furthermore, resveratrol treatment increased the downregulated levels of osteocalcin and Runx2 in MSCs exposed to TNF-α induction. Overexpressed protein levels of NF-κB and β-catenin in TNF-α-induced MSC were downregulated by resveratrol treatment. The results demonstrate that resveratrol improved TNF-α–inhibited osteogenic differentiation of MSC, contributing to slowing osteoporosis progression [87].

In parallel, oxidative stress, a known contributor to bone loss, is another context where resveratrol has demonstrated significant protective action. Data from Feng et al. showed that resveratrol effectively reduced RANKL levels together with tartrate-resistant acid phosphatase 5b levels, but increased OPG levels and attenuated damage to bone microarchitecture, effectively preventing oxidative stress-induced bone loss. Moreover, at the molecular level, the authors confirmed that resveratrol upregulates FoxO1 transcriptional activity by inhibiting the PI3K/AKT signaling pathway, thereby promoting resistance to oxidative damage and limiting osteoclastogenesis. FoxO1 is an important target of resveratrol to exert anti-osteoporotic function, with effects deriving from its ability to enhance redox balance [88].

#### 5.1.3. Curcumin

Among the most studied osteometabolic polyphenols is also curcumin, well known for its anti-inflammatory and antioxidant properties. Notably, Li et al. studied curcumin’s effects on glucocorticoid-induced osteoporosis, a leading cause of secondary osteoporosis. Recently, the “bone–gut axis” theory has linked bone development to intestinal microbial diversity, community composition, and metabolites. Their study demonstrated the efficacy of curcumin in reducing bone loss and restoring bone mineral density, improving trabecular parameters, especially by increasing trabecular number, volume, and thickness and reducing marrow cavity size. Intestinal microbiome sequencing revealed that curcumin treatment improved microbial diversity and altered microbiota composition by increasing beneficial bacteria while decreasing harmful bacteria. Additionally, significant alterations in serum metabolite levels, including raffinose, ursolic acid, spermidine, inosine, hypoxanthine, thiamine, and pantothenic acid, were observed after curcumin treatment. Importantly, these beneficial metabolites and microorganisms were negatively correlated with inflammatory cytokines. In conclusion, curcumin is promising for use against glucocorticoid-induced osteoporosis because it modulates both the intestinal microbiome and serum metabolome while reducing systemic inflammation [89]. Through modulation of the gut microbiota and serum metabolome, curcumin offers a holistic approach to bone regeneration.

Curcumin’s beneficial effect also extends to other pathological contexts, such as diabetes, highlighting its ability to support osteogenesis–angiogenesis coupling. Indeed, curcumin appears to prevent diabetic osteoporosis, a metabolic disease characterized by altered bone microarchitecture and reduced bone mineral density due to hyperglycemia. Osteogenesis–angiogenesis coupling, mediated by MSC, is important in bone formation and regeneration. Results from a 2022 study showed that high glucose impairs the osteogenic and pro-angiogenic capacity of MSC and that curcumin pretreatment restored glucose-induced MSC dysfunction. It was found that inhibition of NF-κB signaling activated by high glucose contributes to curcumin’s protective effects on high glucose-inhibited osteogenesis–angiogenesis coupling in MSC. Based on these findings, the authors suggest that curcumin improved diabetic osteoporosis by restoring coupling between osteogenesis and angiogenesis in MSC under hyperglycemia, partly via inhibition of NF-κB signaling activated by high glucose [90]. The beneficial effect mediated by NF-κB pathway inhibition suggests an interesting mechanism of action even under adverse metabolic conditions.

#### 5.1.4. Other Polyphenols

Beyond the better-known compounds, extracts from traditional plants such as *Tagetes erecta* have revealed interesting osteogenic potential through modulation of specific intracellular signaling pathways. Notably, with minimal side effects, natural remedies based on medicinal plants are increasingly explored for osteoporosis treatment. However, the osteogenic potential of *Tagetes erecta* L. flower, traditionally used for cardiovascular and renal diseases, had not yet been studied. In a 2025 study, authors investigated the osteogenic effects of a polyphenol-enriched extract of T. erecta flowers (TE) and its main components on osteoblast differentiation, focusing on anti-osteoporotic activity. In preosteoblastic MC3T3-E1 cells, TE increased ALP activity, mineralization, and expression of SP7, RUNX2, and ALP. It also induced phosphorylation of GSK-3β at serine 9 and promoted nuclear translocation of β-catenin. Inhibition of β-catenin signaling reversed TE-induced osteogenic effects. Molecular docking suggested strong binding of GSK-3β by TE components, with patuletin showing notable inhibition of GSK-3β activity and enhanced vertebral formation. By targeting GSK-3β and activating β-catenin-mediated pathways, TE appears promising as a new anti-osteoporotic agent. This study highlights TE’s potential for therapeutic use in bone health, justifying further clinical studies to confirm its applicability [91]. Activation of β-catenin pathway and inhibition of GSK-3β by its components suggest a new phytotherapeutic approach for osteoporosis.

Isoflavones, a class of phytoestrogens naturally present in legumes, represent another group of bioactive compounds with favorable effects on bone tissue, particularly under estrogen deficiency conditions. Isoflavones, especially genistein and daidzein, have been shown to stimulate osteoblastic differentiation by increasing RUNX2 expression and BMP-2 signaling, mechanisms involving estrogen receptor activation. Genistein, at 10^−6^ M concentration, significantly increased MC3T3-E1 cell numbers (a murine osteoblast cell line) and ALP activity, confirming its role in promoting bone formation [92]. These effects are consistent with the phytoestrogenic action of isoflavones, which mimic endogenous estrogen activity, particularly relevant in estrogen deficiency. These results reinforce the hypothesis that isoflavones could represent effective support in estrogen-deficient conditions.

The osteoprotective potential of isoflavones has also been confirmed in experimental models with functional legume derivatives, such as the black-eyed pea. The black-eyed pea is a legume with high isoflavone content; authors of a 2019 study focused on the beneficial effects of isoflavones isolated from black-eyed pea in improving osteoporotic condition. MG-63 cells were evaluated for expression of genes involved in BMP-2 signaling, such as BMP-2, OSX, total and phosphorylated Smad 1/5/8 levels, and osteoblast-specific genes, including ALP and OPN. Upon initial exposure to compounds, all marker levels were upregulated. Therefore, results demonstrate that isoflavones isolated from black-eyed pea could be used in the treatment of bone disorders [93]. This evidence supports the potential use of functional legumes in treating bone disorders.

### 5.2. In Vivo Studies

Beyond in vitro studies, numerous preclinical investigations have employed animal models to evaluate the efficacy of polyphenols in preventing or mitigating bone loss associated with osteoporosis. These in vivo studies represent a crucial step to confirm mechanisms observed in vitro and translate them into complex pathophysiological contexts. Experimental animal models allow for a more comprehensive assessment of the osteoprotective activity of phenolic compounds. Analysis of parameters such as BMD, trabecular microarchitecture, biochemical markers of bone turnover, and gene expression in bone tissues has provided significant evidence of the beneficial role of polyphenols (Table 4).

#### 5.2.1. Quercetin

Among the most studied compounds in animal models, quercetin has shown promising results on both osteoblastic and osteoclastic fronts. Yuan et al. investigated the effect of quercetin on promoting proliferation of bone marrow MSC and improving osteoporosis in rats. In vivo, quercetin increased BMD and improved bone biomechanical properties in rat models of postmenopausal osteoporosis. In vitro, TNF-α induced activation of NF-κB and degradation of β-catenin, both significantly inhibited by quercetin. Furthermore, quercetin promoted proliferation and osteogenic differentiation of MSC. In conclusion, quercetin improved in vitro models of osteoporosis and protected against TNF-α-induced impairment of osteogenesis in MSC [94].

Similar findings were reported by Wattel et al., who studied quercetin’s effects on osteoclast differentiation using a murine model with RAW 264.7 cells cultured in the presence of receptor activator of RANKL. Osteoclastogenesis was evaluated by osteoclast number, tartrate-resistant acid phosphatase (TRAP) activity, and bone resorption activity. Quercetin (0.1–10 µM) dose-dependently reduced osteoclastogenesis, with significant effects at low concentrations from 1 to 5 µM. The IC50 was approximately 1 µM. Protein-DNA interaction analysis showed that quercetin pretreatment inhibited RANKL-induced activation of NF-κB and AP-1, transcription factors highly involved in osteoclast differentiation. Their inhibition possibly plays a key role in the reduction in osteoclastogenesis observed with quercetin [61].

The molecular mechanism underlying quercetin’s osteoprotective action has become clearer, partly due to the identification of new mediators such as the long non-coding RNA Malat1. Quercetin upregulates Wnt/β-catenin signaling while inhibiting NF-κB signaling, thereby restoring osteogenesis in MSC compromised by TNFα-induced osteoporosis. Moreover, the functional lncRNA Malat1 has been shown to be a key mediator in quercetin-regulated signaling activities. In an ovariectomy (OVX)-induced osteoporosis mouse model, quercetin administration significantly restored OVX-induced bone loss and structural deterioration. Serum Malat1 levels were also clearly restored after quercetin treatment in the OVX model. Feng et al.’s study concluded that quercetin can restore osteogenesis in TNFα-compromised MSC and osteoporosis-induced bone loss in vivo in a Malat1-dependent manner [95].

Quercetin’s osteoprotective effects extend to extreme environmental conditions such as chronic high-altitude hypoxia (CHH), known to impair skeletal health. In a 2024 study, experimental mice exposed to a simulated 5000 m altitude environment for 8 weeks developed CHH. Results suggest quercetin has significant anti-senescent effects on MSCs and positively impacts the bone marrow microenvironment, supporting its clinical trial as a therapeutic agent for CHH-associated osteoporosis [96].

Additionally, natural quercetin analogs derived from Ulmus wallichiana bark have shown promising osteoprotective effects. Extracts from this bark enhanced peak bone mass in growing rats and preserved trabecular bone mass and cortical bone strength in OVX rats. A novel flavonol-C-glucoside, 6-C-β-D-glucopyranosyl-(2S,3S)-(+)-3′,4′,5,7-tetrahydroxyflavanol (GTDF), isolated from these extracts exhibited non-estrogenic bone-sparing activity in OVX rats, selectively anabolic on osteoblasts without negative effects on other cells. In vitro, GTDF stimulated osteoblast proliferation, survival, and differentiation, without affecting osteoclast or adipocyte differentiation. GTDF treatment in neonatal or adult rats increased mRNA levels of AhR target genes in calvaria or bone marrow stromal cells. In growing female rats, GTDF promoted peak bone accrual parameters in the appendicular skeleton, including increased longitudinal growth, BMD, bone formation rate (BFR), cortical deposition, and bone strength. GTDF enhanced new bone formation to fill defects in femurs of both estrogen-sufficient and deficient rats. In osteopenic OVX rats, GTDF increased BFR and significantly restored trabecular bone compared to intact controls. Overall, data indicate GTDF stimulates osteoblast growth and differentiation via AhR, promotes modeling-driven bone accrual, accelerates bone healing after injury, and exerts anabolic effects in osteopenic rats, likely via direct stimulation of osteoprogenitors [97].

#### 5.2.2. Resveratrol

Resveratrol, another extensively studied polyphenol, has shown promising in vivo results. Osteocytes are recognized as orchestrators of bone remodeling, regulating osteoblast and osteoclast activity. Oxidative stress-induced osteocyte apoptosis plays a crucial role in osteoporosis pathology. Resveratrol, a natural polyphenol, ameliorates postmenopausal osteoporosis. Recent experimental research has focused on the molecular mechanisms underlying resveratrol’s protective effects on osteocytes, particularly under oxidative stress.

A 2023 study constructed an ovariectomy-induced osteoporosis rat model with/without daily intraperitoneal injections of 10 mg/kg resveratrol. Resveratrol mitigated H_2_O_2_-induced osteocyte apoptosis by activating autophagy in MLO-Y4 osteocyte cultures, mediated by dissociation of the Beclin-1/Bcl-2 complex via the AMPK/JNK1-dependent metabolic pathway, thus regulating osteocyte function. Additionally, resveratrol treatment reduced osteocyte oxidative stress, inhibited osteocyte apoptosis, and promoted autophagy in ovariectomized rats. The study suggests resveratrol protects against oxidative stress by restoring osteocyte autophagy and alleviating apoptosis through AMPK/JNK1 activation, dissociating Bcl-2 from Beclin-1 [98].

Further studies confirm resveratrol’s efficacy against osteoporosis via different molecular pathways and animal models. Song et al. studied resveratrol’s benefits in an ovariectomized rat model, using eosin staining to examine femoral trabecular tissue. They found miR-193a overexpressed and SIRT7 downregulated in osteoporosis. Resveratrol suppressed miR-193a to promote osteogenic differentiation. Mechanistically, miR-193a negatively regulated SIRT7, which in turn promoted osteogenic differentiation via NF-κB signaling. Resveratrol’s beneficial function was mediated via NF-κB signaling modulated by miR-193a/SIRT7 to alleviate osteoporosis in vivo. This presents a novel perspective for osteoporosis diagnosis and treatment [99].

Additional research explored resveratrol’s effects in genetically and age-related altered animal models. Senescence-accelerated mice with defective osteoblastogenesis and accelerated skeletal aging were used to assess resveratrol’s effects. In vivo application of 100 mg/kg intraperitoneal resveratrol every other day for 2 months improved bone formation and counteracted accelerated bone loss. Resveratrol restored osteogenic decline during MSC senescence by rescuing mitochondrial function and promoting mitochondrial autonomous gene transcription. The polyphenol upregulated mitofilin, a key component of mitochondrial contact sites and cristae organizing systems essential for mitochondrial homeostasis and MSC osteogenesis; its deficiency leads to MSC senescence and bone loss. These findings reveal critical osteogenic functional improvements in senescent MSC, identifying mitofilin as a novel mechanism mediating resveratrol’s promotion of mitochondrial function in stem cell senescence [100].

Beyond postmenopausal and senescent models, recent research investigated resveratrol’s application in secondary osteoporosis conditions like spinal cord injury (SCI)-induced osteoporosis. A 2025 study showed resveratrol enhances protective effects of calcium supplements against SCI-induced osteoporosis via the SIRT1/FOXO3a pathway regulating bone metabolism. SCI often leads to osteoporosis due to immobilization and hormonal imbalance. Calcium supplementation helps maintain bone health but can have limited efficacy. Using a murine SCI model, combined resveratrol and calcium preserved bone mass, microarchitecture, strength, and fracture resistance synergistically compared to calcium alone post-SCI. This was accompanied by increased osteoblastic markers, reduced osteoclastic markers, and enhanced SIRT1/FOXO3a expression and activation. The results suggest resveratrol potentiates calcium’s bone protective effects in SCI-induced osteoporosis by modulating SIRT1/FOXO3a signaling and osteoblast/osteoclast activity, presenting a promising therapeutic approach [101].

#### 5.2.3. Fisetin

Although resveratrol has been extensively studied, other phenolic compounds, including flavonoids like fisetin, have shown significant potential for osteoporosis treatment via innovative mechanisms. Yousefzadeh et al. evaluated a series of flavonoid polyphenols for senolytic activity using murine fibroblasts. The most effective senotherapeutic flavonoid, fisetin, was tested in progeroid and aged wild-type mice to determine effects on senescence markers, age-related histopathology, disease markers, health span, and lifespan. Acute or intermittent fisetin treatment reduced senescence markers in various tissues, with cell-type specificity observed in murine and human adipose tissue. Fisetin administration in aged wild-type mice restored tissue homeostasis, reduced age-related pathologies, and extended median and maximal lifespan [102].

Fisetin’s role in supporting osteoblastogenesis was further highlighted in inflammatory osteoporosis models, where it showed protective effects on osteoblast precursors. In vivo, LPS-treated mice exhibited osteoporotic traits with severe repression of osteoblastic markers. In this model, fisetin partially counteracted transcriptional inhibition of osteocalcin and type I collagen alpha 1. Mechanistic analysis revealed fisetin’s ability to regulate runt-related transcription factor 2, a key organizer of osteoblast development and maturation [103].

#### 5.2.4. EGCG

Among promising natural compounds, EGCG, the major green tea catechin, has demonstrated significant bone-protective effects. EGCG showed protective effects against secondary osteoporosis in a murine model, particularly via Wnt/β-catenin signaling. Treatment with EGCG significantly reduced serum calcium, urinary calcium, body weight, and fat mass, while increasing leptin levels in secondary osteoporosis mice. EGCG significantly inhibited articular cartilage and trabecular bone structural scores in proximal tibial metaphysis. It also reduced alkaline phosphatase activity significantly and induced a marked increase in protein expression of cyclin D1, Wnt, and β-catenin [104].

EGCG’s potential was further confirmed in murine arthritis models, suggesting a dual role in modulating inflammation and bone resorption. Morinobu et al. evaluated EGCG’s effects on osteoclast differentiation and experimental arthritis in mice. Arthritis was induced by injecting a collagen monoclonal antibody cocktail. EGCG (20 µg/g body weight) was administered intraperitoneally daily from day 0 to 15. EGCG reduced multinucleated TRAP-positive cell generation, bone resorption activity, and osteoclast-specific gene expression without affecting cell viability. EGCG reduced nuclear factor of activated T cells c1 (NF-ATc1) expression, but not NF-kappaB, c-Fos, or c-Jun, suggesting NF-ATc1 reduction as a molecular basis for EGCG action. EGCG also improved clinical symptoms and reduced histological scores in arthritic mice. However, the in vivo effect on osteoclast differentiation was unclear, possibly due to EGCG’s inflammation suppression. In conclusion, EGCG suppressed osteoclast differentiation and improved experimental arthritis in mice short term [105].

#### 5.2.5. Other Polyphenols

Finally, alongside classic polyphenols, their gut microbiota-derived metabolites are emerging as new bioactive agents in osteoporosis context. Intracellular ROS accumulation is key for osteoclast differentiation. Plant-derived polyphenols that reduce ROS production have been widely studied for osteoporosis treatment. However, these compounds are rarely absorbed in the small intestine, instead converted into low-molecular-weight phenolic acids by colon microbiota. These phenolic acids can then be absorbed systemically. 4-Hydroxyphenylacetic acid (4-HPA) is a major metabolite generated by human gut microbiota. A 2024 study clarified 4-HPA’s role in osteoclastogenesis and osteoporosis treatment. 4-HPA inhibited osteoclast differentiation and function, and reduced osteoclast-specific gene expression. Additionally, 4-HPA reduced ROS accumulation by regulating Nrf2 and subsequently inhibiting NF-κB and MAPK pathways. Finally, a murine ovariectomized osteoporosis model showed 4-HPA effectively prevented bone loss. The study revealed that 4-HPA, a polyphenol metabolite produced by gut microbes, inhibits osteoclast formation and treats osteoporosis, providing a new experimental foundation and candidate drug for osteoporosis treatment [106].

## 6. Clinical Studies

Beyond in vitro studies and animal models, although promising results have been obtained, their translation into clinical practice requires more robust evidence. In this context, in vivo human studies are fundamental to understanding the actual efficacy and safety of bioactive compounds, such as polyphenols, in the prevention and treatment of chronic conditions like osteoporosis. However, clinical literature on the topic remains limited, making these studies a priority for future research. Therefore, it is necessary to further investigate the potential of polyphenols through clinical trials that can validate preclinical observations and provide applicable therapeutic guidelines.

Although preclinical data have outlined an encouraging picture, it is essential to understand their true translational relevance through studies conducted in humans. To confirm the biological validity observed in experimental models, several clinical studies have explored the effects of polyphenols in the human population.

Among the most clinically investigated compounds, resveratrol stands out for its documented osteoprotective activity. In particular, in vitro and in vivo studies in rodent models indicate a protective role of resveratrol for bones, especially in ovariectomized rat models that mimic postmenopausal osteoporosis caused by estrogen deficiency. Hypothesizing a circulatory benefit of resveratrol in bone tissue, authors have studied whether resveratrol supplementation could improve bone health in postmenopausal women. A relevant example in this field is represented by a long-term clinical study investigating the efficacy of resveratrol as a dietary supplement. This randomized, double-blind, placebo-controlled crossover clinical trial, lasting 24 months, was conducted to evaluate the effects of resveratrol (75 mg twice daily) on cognitive functions, cerebrovascular functionality, bone health, cardiometabolic markers, and wellbeing in postmenopausal women. After 12 months of resveratrol supplementation compared with placebo, positive effects on bone density in the lumbar spine and femoral neck were observed, accompanied by a 7.24% reduction in type 1 collagen C-terminal telopeptide levels, a marker of bone resorption, relative to placebo. The increase in femoral neck bone mineral density resulted in improved T-score and reduced 10-year probability of major fracture and hip fracture risk. The study also revealed that the bone protective benefit of resveratrol was greater among participants who supplemented with vitamin D and calcium [107].

Beyond isolated use, the synergistic association of resveratrol with other bioactive compounds, such as equol derived from fermented soy, has also been explored. Estrogen deficiency is one of the main causes of bone mineral density loss in postmenopause. A 2023 study aimed to evaluate the effects of equol and resveratrol on bone turnover biomarkers in postmenopausal women. Sixty healthy postmenopausal women were randomly assigned to receive 200 mg fermented soy containing 10 mg equol and 25 mg resveratrol. Overall, the intervention significantly increased whole-body bone mineral density compared to placebo. These data indicate that the combination of equol and resveratrol can positively modulate bone turnover biomarkers, suggesting resveratrol as a potential approach to prevent age-related bone loss in postmenopausal women [108].

Similarly, other dietary polyphenols have demonstrated therapeutic potential in bone health contexts, such as blueberries. In a dose–response study on blueberries, using urinary calcium tracers from pre-labeled bones to reflect changes in bone balance, authors hypothesized that blueberry consumption would reduce bone loss dose-dependently. Fourteen healthy non-osteoporotic women, ≥4 years postmenopause, were treated with 50 nCi Ca41, a long-lived radioisotope, and maintained in equilibrium for 5 months for Ca41 deposition in the bone. After a 6-week baseline, participants were assigned randomly to a sequence of three 6-week interventions: low (17.5 g/day), medium (35 g/day), or high (70 g/day) doses of freeze-dried blueberry powder equivalent to 0.75, 1.5, or 3 cups of fresh blueberries incorporated in foods and beverages. Data show that in postmenopausal women, blueberry interventions improved net bone calcium balance at lower doses but not at higher doses. Specifically, net bone calcium retention increased by 6% with the low dose and 4% with the medium dose compared to no treatment. Urinary hippuric acid excretion increased dose-dependently with blueberry consumption. No significant associations were found between bone resorption biomarkers, 25-hydroxyvitamin D, and interventions. The authors propose that moderate blueberry intake (<1 cup/day) may be an effective strategy to mitigate bone loss in healthy postmenopausal women [109].

Supporting this line of research, epidemiological data on dietary flavonoid intake are also noteworthy. These data fit into a broader context suggesting a link between flavonoid intake and bone health. Dietary flavonoid intake has been associated with improved bone health markers in U.S. cohorts. Wang et al.’s research team aimed to assess whether dietary flavonoid intake was associated with BMD, BMC and bone area in the lumbar spine and femoral neck in a nationally representative sample of middle-aged and older U.S. adults. Cross-sectional data from individuals aged 50 years and older were used. However, this analysis provides contradictory evidence, indicating that the association between flavonoids and bone health is not yet fully clear. Therefore, further longitudinal and interventional studies are needed to confirm these data [110].

Among the most studied dietary sources in this context, green tea has attracted attention for its high catechin content, especially EGCG, and its potential beneficial effects on bone. Tea consumption may be beneficial for osteoporosis due to its antioxidant capacity. However, the lack of objective data characterizing tea consumption has hindered precise evaluation of the association between tea intake and bone mineral density. Therefore, a study by Li Shen et al. was designed to test the feasibility of an intervention including green tea polyphenols (GTPs) in postmenopausal women with osteopenia. Specifically, 140 postmenopausal women with osteopenia received 500 mg GTP daily for 24 weeks. The authors evaluated a new complementary and alternative medicine strategy involving dietary supplementation to alleviate bone loss in osteopenic postmenopausal women [111].

Dried plums, functional foods rich in phenolic compounds, have also been investigated for their impact on bone metabolism. Data from human studies suggest that dried plum intake may improve lipid metabolism, anti-inflammatory and antioxidant defense systems, affecting cardiovascular health. For this reason, Al-Dashti et al. tested the hypothesis that short-term consumption of low and moderate levels of dried plums increases bone resorption and vascular function. In the study, twenty-seven healthy postmenopausal women were randomly assigned to consume either six dried plums (~42 g) or two dried plums (~14 g) daily for 2 weeks. The results suggest a potentially favorable impact of dried plums on bone health, though the short study duration requires confirmation in larger studies [112].

In summary, currently available clinical evidence, although still limited, supports the hypothesis that dietary polyphenols may exert a protective effect on human bone, particularly in postmenopausal women (Table 5).

## 7. Discussion

This comprehensive analysis of the current scientific literature underscores the therapeutic potential of polyphenols in the prevention and management of osteoporosis, a chronic, multifactorial disease characterized by an imbalance between bone formation and resorption. Given the high prevalence of osteoporosis, particularly among postmenopausal women, and the limitations associated with conventional pharmacological therapies, including adverse effects, high costs, and poor long-term adherence, there is a growing interest in complementary or alternative strategies grounded in natural compounds [107,108].

Preclinical studies consistently demonstrate that polyphenols positively influence bone metabolism through a wide range of mechanisms. These include antioxidant activity, inhibition of pro-inflammatory cytokines, modulation of osteogenic signaling pathways (e.g., Wnt/β-catenin, MAPKs, BMPs), and regulation of the RANKL/RANK/OPG axis, which is crucial for osteoclastogenesis [61]. Notably, compounds such as quercetin, resveratrol, curcumin, and isoflavones exhibit dose-dependent effects: while low concentrations tend to stimulate osteoblast differentiation and activity, high concentrations may induce cytotoxicity or shift MSC toward adipogenic differentiation. These observations highlight the importance of identifying optimal therapeutic windows [70,84,99].

Quercetin, for example, has shown significant osteogenic and antioxidant properties in vitro and in vivo, as well as selective antitumor effects on osteoblastic cancer cells. Resveratrol has demonstrated protective effects under inflammatory and senescent conditions via pathways such as AMPK, SIRT1, and FoxO1, contributing to the inhibition of osteocyte apoptosis and the promotion of bone formation [84]. Curcumin and EGCG have shown additional benefits by modulating gut microbiota, suggesting a potential therapeutic role for the gut–bone axis [90]. Emerging evidence also points to the bioactivity of polyphenol-derived microbial metabolites, such as 4-hydroxyphenylacetic acid, which inhibit osteoclastogenesis and mitigate bone loss in experimental models [106].

On the translational side, although human clinical data remain limited, the available studies provide encouraging results. Polyphenol supplementation, particularly with resveratrol, has been associated with improved bone mineral density and reductions in bone resorption markers in postmenopausal women. Moreover, clinical trials involving dietary sources of polyphenols, such as blueberries, green tea, and dried plums, have reported favorable outcomes on bone calcium retention and turnover, further supporting the hypothesis of a protective role [108,109].

Beyond polyphenols, other classes of plant-derived metabolites have also shown promising activity in osteoporosis. Terpenoids (such as ginsenosides from *Panax ginseng* and icariin from *Epimedium brevicornum*) exert anabolic effects on bone by stimulating osteoblast differentiation and inhibiting osteoclastogenesis through estrogen receptor-mediated and MAPK-related pathways [113,114]. Alkaloids, including berberine, have demonstrated anti-resorptive properties by suppressing osteoclast activity and modulating oxidative stress [115,116]. In addition, lignans and saponins are emerging as potential regulators of bone metabolism, although their mechanisms remain less well defined compared to polyphenols [117]. These findings suggest that polyphenols are part of a broader spectrum of phytochemicals with potential utility in osteoporosis prevention and management.

With regard to drug development, current trends point toward the structural optimization of natural polyphenols and the synthesis of chemical analogs with improved pharmacokinetics and stability. Resveratrol derivatives, curcumin analogs, and synthetic isoflavones are under investigation for their enhanced bioactivity and reduced metabolic degradation. Nanotechnology-based formulations, including liposomes, polymeric nanoparticles, and micelles, are being tested to overcome poor oral bioavailability and achieve sustained release. Despite these advances, no polyphenol-derived compound has yet received regulatory approval as a specific anti-osteoporotic drug. However, isoflavone-based nutraceuticals and supplements are already commercially available and marketed for bone health in postmenopausal women, although they are positioned as dietary supplements rather than pharmaceutical drugs. This highlights the translational gap between experimental progress and full clinical validation.

Nonetheless, several challenges must be addressed before polyphenols can be routinely used in clinical practice. First, their poor oral bioavailability, due to low solubility, rapid metabolism, and individual variability in gut absorption, represents a major limitation. Interactions with dietary components and the gut microbiome further complicate pharmacokinetics [55]. Second, the lack of standardization in polyphenol formulations, dosage, and study design hinders the reproducibility and comparability of clinical outcomes. Finally, variability in individual responses, shaped by genetic, epigenetic, metabolic, and microbial factors, demands a more personalized approach.

Future research should focus on the development of novel delivery systems (e.g., nanoparticles, bioactive carriers) to enhance systemic bioavailability, as well as on identifying reliable biomarkers of efficacy. Integrating omics technologies (metabolomics, transcriptomics, microbiomics) may clarify systemic interactions between polyphenols and bone health, paving the way for precision nutraceutical strategies.

In conclusion, polyphenols offer a promising avenue in the integrative management of osteoporosis. Their inclusion in multimodal strategies, combining lifestyle modification, dietary interventions, and pharmacological support, has the potential to improve bone health in both general and high-risk populations. A translational, interdisciplinary approach will be essential to fully exploit the therapeutic potential of polyphenols and bridge the gap between experimental evidence and clinical application.

## Figures and Tables

**Table 1:** Classification of polyphenols, representative examples, and their main dietary sources.

Polyphenol Class	Subcategories	Examples	Main Dietary Sources
Flavonoids	Flavonols, Flavones, Isoflavones, Anthocyanins, etc.	Quercetin, Genistein	Green tea, red wine, soy, citrus, apple
Phenolic Acids	Derivatives of benzoic and cinnamic acids	Caffeic acid, Ferulic acid	Coffee, whole grains, fruit
Stilbenes	—	Resveratrol	Grapes, red wine, peanuts
Lignans	—	Secoisolariciresinol	Flaxseeds, cereals, legumes

**Table 2:** Molecular mechanisms of key polyphenols, including their actions, signaling pathways, and effects on bone cells.

Polyphenol	Key Molecular Actions	Pathways Involved	Effects on Bone Cells
Quercetin	↑ ALP, ↑ RUNX2, ↓ ROS	MAPKs, ERK, NF-κB	Promotes osteoblasts, inhibits osteoclasts
Resveratrol	↑ SIRT1, ↑ FoxO1, ↓ p53	PI3K/AKT, MDM2/p53	Anti-apoptotic, anti-inflammatory
Curcumin	↑ Wnt/β-catenin, ↑ microbiota	NF-κB, BMP-2	Enhances bone mineralization
EGCG	↓ NF-ATc1, ↓ TRAP	Wnt/β-catenin, MAPKs	Reduces osteoclastogenesis
Isoflavones	↑ ERβ binding, ↑ BMP-2	ER-mediated, SMAD	Estrogen-like effects

**Table 3:** In vitro experiments: main effects observed and molecular mechanisms.

Polyphenol/Extract	Experimental Model	Main Observed Effects	Molecular Mechanisms Involved
Quercetin	Osteoblasts in vitro	↑ ALP (early osteogenesis marker)	ERK activation downstream of estrogen receptor
	Osteoblasts in vitro	↑ Osteoblast differentiation	↑ TGF-β1, BMP-2, RUNX2; activation of MAPKs (ERK1/2, p38, JNK)
	Osteoblasts in vitro	↑ Viability, adhesion, migration; protective effect	Safe on healthy cells; at high concentrations (20–100 μM) ↓ viability of osteoblastic tumor cells
	Osteoblasts exposed to cigarette smoke	↑ Viability; protection against oxidative stress	↑ HO-1, SOD; reduction in ROS-induced damage
	Osteoblasts exposed to LPS	Restores LPS-inhibited differentiation	MAPK pathway activation; protection from infection-related osteolysis
	MC3T3-E1	↑ ALP, mineralized nodules; ↓ apoptosis and ROS	↑ Runx2, Osterix, Bcl-2; ↓ Caspase3, Bax
	Murine MSC	↑ Proliferation and osteogenic differentiation	↑ BMP pathways, OSX, RUNX2, OPN
	MSC	↓ ALP, mineralization; ↑ adipogenesis	↓ ALPL, Col1a1, OCN; dose-dependent and potentially adverse effects
	Osteoblasts from fetal rat calvaria	↑ HO-1, GCLC; ↓ pERK1/2, NF-κB	Variable antioxidant effect; no synergy among metabolites
Quercetin + biomaterials	Silk fibroin/HA scaffold with quercetin	↑ Osteogenesis and bone regeneration	Creation of pro-osteoblastic biomimetic microenvironment
Quercetin (nanoformulations)	Hydrogel + quercetin	↑ Cellular uptake, ↑ bone regeneration	↑ Bioavailability and stability; promising for orthopedic applications
Resveratrol	MSC	↑ Osteogenic differentiation	MDM2/p53, NF-κB/β-catenin pathways; protective effect
	MSC	Protects differentiation under inflammation	↓ NF-κB, β-catenin; ↑ Runx2, osteocalcin
	Oxidative stress models	↓ RANKL, TRAP5b; ↑ OPG	↑ FoxO1 via PI3K/AKT inhibition; ↓ osteoclastogenesis
Curcumin	Glucocorticoid-induced osteoporosis	↑ BMD, trabeculae, microbiota and beneficial metabolites	Gut–bone axis modulation; ↓ inflammatory cytokines
	MSC under hyperglycemia	Restores osteogenesis–angiogenesis coupling	NF-κB inhibition under glucose-induced stress
*Tagetes erecta* (extract)	MC3T3-E1	↑ ALP, mineralization, RUNX2, SP7	β-catenin activation, GSK-3β inhibition; patuletin effective
Isoflavones	MC3T3-E1	↑ ALP, osteoblast proliferation	Estrogen receptor activation; ↑ RUNX2, BMP-2
	MG-63	↑ BMP-2, OSX, Smad1/5/8, ALP, OPN	Supports functional legumes as anti-osteoporotic agents

**Table 4:** In vivo experiments: main effects observed and molecular mechanisms.

Polyphenol/Extract	Animal Model	Main Observed Effects	Molecular Mechanisms Involved
Quercetin	Rats, postmenopausal osteoporosis	↑ BMD, improved bone biomechanical properties	Inhibits TNF-α-induced NF-κB activation, preserves β-catenin; promotes MSC proliferation and osteogenic differentiation
	Murine RAW 264.7 cells + RANKL	↓ Osteoclastogenesis, dose-dependent	IC50 ~1 µM; inhibits NF-κB and AP-1 activation, reduces osteoclast differentiation
	OVX mice	Restores OVX-induced bone loss and structural deterioration	Upregulates Wnt/β-catenin, inhibits NF-κB; effect mediated by lncRNA Malat1
	Mice, chronic high-altitude hypoxia	Anti-senescent effects on MSC, improved bone marrow microenvironment	Potential therapeutic for CHH-associated osteoporosis
Quercetin analogs (GTDF)	OVX and growing rats	↑ Peak bone mass, trabecular/cortical bone strength, BFR	Stimulates osteoblast proliferation, survival, differentiation via AhR; non-estrogenic, selective anabolic effect
Resveratrol	OVX rats, H_2_O_2_-treated osteocytes	↓ Osteocyte apoptosis, promotes autophagy	AMPK/JNK1 pathway; dissociation of Beclin-1/Bcl-2 complex
	OVX rats, femoral trabecular tissue	↑ Osteogenic differentiation	Suppresses miR-193a, upregulates SIRT7, modulates NF-κB signaling
	Senescence-accelerated mice	↑ Bone formation, counters accelerated bone loss	Restores mitochondrial function, upregulates mitofilin, improves MSC osteogenesis
	SCI-induced osteoporosis in mice	Preserved bone mass, strength, microarchitecture	SIRT1/FOXO3a pathway; ↑ osteoblastic markers, ↓ osteoclastic markers
Fisetin	Progeroid/aged mice	↓ Senescence markers, extended health span & lifespan	Senolytic activity, tissue-specific, restores tissue homeostasis
	LPS-induced inflammatory osteoporosis in mice	↑ Osteoblastic markers (OCN, Col1a1)	Regulates RUNX2 to support osteoblast development and maturation
EGCG	Secondary osteoporosis murine model	↓ Serum & urinary calcium, ↑ leptin, improved trabecular structure	Activates Wnt/β-catenin signaling; inhibits bone resorption, ↑ cyclin D1
	Murine experimental arthritis	↓ TRAP-positive osteoclasts, ↓ bone resorption	↓ NF-ATc1 expression; modulates inflammation; protects against osteoclast differentiation
4-HPA	OVX murine osteoporosis model	Prevented bone loss, ↓ osteoclast differentiation	Regulates ROS, Nrf2 activation, inhibits NF-κB and MAPK pathways; gut microbiota-derived polyphenol metabolite

**Table 5:** Summary of clinical studies evaluating the efficacy of polyphenols in osteoporosis, detailing populations, outcomes, duration, and study types.

Polyphenol Studied	Population	Main Findings	Study Type
Resveratrol	Postmenopausal women	↑ BMD, ↓ CTX	RCT, double-blind
Curcumin	Patients with GIOP	↑ trabecular bone, ↑ beneficial microbiota	Controlled study
EGCG	Women with osteopenia	Modest increase in BMD	Intervention trial

## Data Availability

The original contributions presented in this study are included in the article. Further inquiries can be directed to the corresponding authors.

## Abbreviations

The following abbreviations are used in this manuscript:

4-HPA	4-Hydroxyphenylacetic acid
AP-1	Activator protein 1
ALP	Alkaline phosphatase
BMD	Bone mineral density
BMP	Bone morphogenetic protein
CHH	Chronic high-altitude hypoxia
EGCG	Epigallocatechin gallate
MSC	Mesenchymal stem cells
MAPKs	Mitogen-Activated Protein Kinases
NF-κB	Nuclear factor kappa B
OPG	Osteoprotegerin
OSX	Osterix
OVX	Ovariectomy
ROS	Reactive oxygen species
RANKL	Receptor Activator of Nuclear Factor κB Ligand
RUNX2	Runt-related transcription factor 2
SCI	Spinal cord injury
SOD	Superoxide dismutase
TNF-α	Tumor necrosis factor-alpha
